# “I cannot see your fear!” Altered recognition of fearful facial expressions in anorexia nervosa

**DOI:** 10.3389/fpsyg.2023.1280719

**Published:** 2023-12-06

**Authors:** Giulia Vaioli, Ilaria Bastoni, Valentina Villa, Leonardo Mendolicchio, Gianluca Castelnuovo, Alessandro Mauro, Federica Scarpina

**Affiliations:** ^1^I.R.C.C.S. Istituto Auxologico Italiano, U.O. di Neurologia e Neuroriabilitazione, Ospedale San Giuseppe, Piancavallo, Italy; ^2^I.R.C.C.S. Istituto Auxologico Italiano, Laboratorio di Psicologia, Ospedale San Giuseppe, Piancavallo, Italy; ^3^I.R.C.C.S. Istituto Auxologico Italiano, U.O. dei Disturbi del Comportamento Alimentare, Ospedale San Giuseppe, Piancavallo, Italy; ^4^Psychology Department, Università Cattolica del Sacro Cuore, Milan, Italy; ^5^“Rita Levi Montalcini” Department of Neurosciences, University of Turin, Turin, Italy

**Keywords:** anorexia nervosa, facial emotion recognition, fear, anger, alexithymia

## Abstract

**Background:**

The evidence about facial emotion recognition in anorexia nervosa as the role of alexithymic traits on this emotional ability is conflicting and heterogeneous.

**Objective:**

We assessed the capability of recognizing facial expressions of two primary emotions, fear, and anger, in the context of anorexia nervosa.

**Methods:**

Women affected by anorexia nervosa were compared with healthy weight women in a well-established implicit facial emotion recognition task. Both reaction time and level of accuracy were computed. Moreover, the individual levels of alexithymia were assessed through a standard self-report questionnaire.

**Results:**

Participants with anorexia nervosa reported a significantly lower performance in terms of reaction time and accuracy when the emotion of fear—but not anger—was the target. Notably, such an alteration was linked to the levels of alexithymia reported in the self-report questionnaire.

**Conclusion:**

In anorexia nervosa, difficulties in processing facial fearful (but not angry) expressions may be observed as linked to higher expressions of alexithymic traits. We suggested future research in which emotional processing will be investigated taking into account the role of the bodily dimensions of emotional awareness.

## Introduction

Previous evidence relative to facial emotion recognition ability in anorexia nervosa (AN) reported in the literature is conflicting. Some studies pointed out a higher difficulty in recognizing others’ emotions (Kucharska-Pietura et al., 2004; Castro et al., 2010; Dapelo et al., 2016; Blomberg et al., 2021), and specifically disgust (Lulé et al., 2014; Dapelo et al., 2016), fear (Kucharska-Pietura et al., 2004; Lulé et al., 2014), and sadness (Kucharska-Pietura et al., 2004). Other studies did not report an altered performance (Mendlewicz et al., 2005; Kessler et al., 2006; Lulé et al., 2014; Gramaglia et al., 2016; Wyssen et al., 2019; Duriez et al., 2021) or—counterintuitively—described individuals with AN as being faster (Lulé et al., 2014) and highly accurate in recognizing facial expressions of anger (Dapelo et al., 2016), disgust (Lulé et al., 2014; Duriez et al., 2021), fear (Sfärlea et al., 2016), and sadness (Duriez et al., 2021), with only one study reporting a higher accuracy in recognizing happiness (Lulé et al., 2014). This heterogeneity can be due to the variability between studies in terms of the clinical characteristics of the enrolled participants, such as disease severity and duration, as well as the presence of psychiatric comorbidities. Moreover, inter-individual differences in cognitions and attitudes toward emotions may have a confounding and not-experimentally controlled effect on performance in emotion-related paradigms (Scarpina et al., 2018; Vaioli and Scarpina, 2021). This is the case with the very traditional facial emotion recognition task, in which individuals are asked to label the emotions shown by their faces. This criticism may be of particular importance in the context of AN: this syndrome is characterized by higher levels of subjective self-control (Casper et al., 1992; Vitousek and Manke, 1994; Butler and Montgomery, 2005), which may affect performance in very explicit tasks. Because of this methodological consideration, implicit versions of the facial emotion recognition task might be preferable, as they decrease the role played by volition and behavioral control (Vaioli and Scarpina, 2021). However, all the previous evidence reported in the literature about the ability to recognize emotions in AN relies on the explicit, and not the implicit version, of the facial emotion recognition task.

This cross-sectional experimental study aimed to explore the recognition of facial emotional expressions in AN, adopting a well-established implicit behavioral paradigm (Scarpina et al., 2018, 2021, 2022). In this task, participants are asked to detect and recognize (i.e., they have to provide an answer when they see the target), but not to label (as done in the other experiments exploring facial emotion recognition in AN), the emotion shown by faces. Notably, in this research, individual performance was analyzed, taking into account the phenomenon of redundant target effect (Miniussi et al., 1998; Tamietto et al., 2006, 2007), according to which the detection and recognition of a visual (even emotional) target is enhanced in the case of simultaneous events but reduced in the case of competitive ones. Because of the psychophysical nature of this phenomenon, the role of volition in performance has decreased. As done in previous articles (Scarpina et al., 2018, 2021, 2022), the two primary emotions of fear and anger were tested. Crucially, some previous evidence reported individuals with AN being more accurate than healthy controls for both the emotions of anger (Dapelo et al., 2016) and fear (Sfärlea et al., 2016). Thus, we may expect to observe a higher performance for both emotions when their recognition is tested implicitly. Nevertheless, considering the other piece of evidence suggesting an overall lower level of accuracy for negative emotions in AN (Kucharska-Pietura et al., 2004; Dapelo et al., 2016; Blomberg et al., 2021), we cannot exclude the opposite scenario with a lower performance in our participants with AN.

Two previous studies (Kessler et al., 2006; Lulé et al., 2014) investigated the ability to recognize facial emotions in AN, taking into account the individual levels of expression of alexithymic traits. Indeed, the levels of alexithymia (Taylor et al., 1997) may interact with facial emotion recognition (Parker et al., 1993; Ihme et al., 2014; Jongen et al., 2014; Scarpazza et al., 2015; Maier et al., 2016; Fujiwara, 2018; Scarpazza et al., 2018; Starita et al., 2018), especially in the case of anger and fear (Prkachin et al., 2009), affecting social cognition abilities (Starita et al., 2018). The results of these previous studies are puzzling. Kessler et al. (2006) did not report any differences between participants with AN and healthy individuals in the facial emotion recognition ability; on the other hand, Lulé et al. (2014) observed that their participants with AN recognized negative emotions less accurately than controls, while they were more accurate in recognizing positive emotions. Moreover, the authors observed shorter reaction times (i.e., suggesting faster recognition) for all the facial stimuli. Crucially, even though the two studies reported different behavioral results, both were in agreement in reporting no significant relationship with the level of alexithymia. Some methodological considerations could be made about these two previous studies (Kessler et al., 2006; Lulé et al., 2014): they investigated facial recognition of the basic emotions (anger, fear, sadness, happiness, surprise, and disgust), all tested in the same experimental task. Moreover, they used a correlational approach to assess the relationship between the experimental behavior and the level of alexithymia, measured through self-report questionnaires. In this study, we aimed to provide new evidence in this debate and specifically to verify if the ability to recognize facial emotions, such as anger and fear, in our sample of female individuals with AN may be linked to the level of alexithymic traits when measured with the self-report Toronto Alexithymia Scale - 20 (Bagby et al., 1994). We adopted a more stringent statistical approach, in which the level of alexithymia was included as a covariate in the statistical model rather than using a correlational approach. According to some previous studies (Kessler et al., 2006; Lulé et al., 2014), we may expect higher alexithymic traits in participants with AN, but without a clear *a priori* hypothesis about its role in explaining the possible difference between groups in detecting (i.e., reaction time) and recognizing (i.e., the level of accuracy) the two tested emotions.

## Methods

This study was approved by the Ethical Committee of the Istituto Auxologico Italiano, IRCCS, Milan, Italy (ID 21C306). Participants gave informed written consent before taking part in the study and were volunteers. They were free to withdraw at any point during the study and were naïve to the rationale of the study.

### Participants

Right-handed (cisgender) females were enrolled, since AN is largely prevalant in women. Moreover, emotional experience and expression differ between genders (Brody and Hall, 1993; Bruzzese et al., 2019). Our participants were consecutively recruited at admission to the hospital before receiving treatment for an eating disorder. Participants were included in this study if they satisfied the Diagnostic and Statistical Manual of Mental Disorders, Fifth Edition’s criteria for anorexia nervosa (i) restriction of food intake leading to weight loss or a failure to gain weight resulting in a “significantly low body weight” of what would be expected for someone’s age, sex, and height; (ii) fear of becoming fat or gaining weight; (iii) having a distorted view of themselves and of their condition). Both “restricting type” and “binge-eating/purging type” (American Psychiatric Association, 2013) were included. The exclusion criteria were: less than 18 years old; the presence or history of a neurological or severe psychiatric disorder, according to an expert psychiatrist examination. An assessment of the eating style was performed as part of the clinical routine through the Eating Disorder Inventory™–3 (Garner, 2004), which showed satisfactory internal consistency and validity (Giannini et al., 2008), and the Binge Eating Scale (Gormally et al., 1982), about which evidence about its statistical properties in discriminating clinically significant cases of binge eating is reported in the literature [i.e., (Duarte et al., 2015; Imperatori et al., 2016)]. Moreover, the self-reported severity of psychopathology and the level of psychological well-being were measured through the Symptom Checklist-90 (Derogatis, 1977), about which the statistical properties are largely described in Derogatis and Unger (Derogatis and Unger, 2010), and the Italian version (Grossi et al., 2006) of the Psychological General Wellbeing Index (Dupuy, 1984), which is largely used in clinical and not clinical settings considering its high statistical properties (Grossi et al., 2006). All the psychological questionnaires were scored according to the normative articles.

In this study, we included data about a group of healthy weight women as controls, extracted randomly from a previous database relative to the same task (Scarpina et al., 2018, 2021), according to the following exclusion criteria: eating disorders; neurological or psychiatric disorders, including clinical depression and anxiety disorders; under medical treatments in the previous 3 months; a level of BMI below or over the healthy range (18.5—24.9).

### The level of alexithymic traits

All participants completed the Italian version (Bressi et al., 1996) of the Toronto Alexithymia Scale - 20 (Bagby et al., 1994). Specifically, this questionnaire describes the overall self-report level of expression of the alexithymic trait, specifically in terms of difficulties in identifying feelings, difficulties in describing feelings, and externally oriented thinking. The questionnaire had acceptable internal consistency (α = 0.81); the test–retest reliability was 0.77.

### Facial emotion recognition task

We borrowed the experimental task from previous studies investigating facial emotion recognition in clinical diseases (Scarpina et al., 2018, 2021, 2022). In the task, we presented pictures representing male and female faces with an emotional expression (i.e., the target, thus it can be an expression of fear or anger) (Ekman and Friesen, 1976) in four experimental conditions: (i) *unilateral*, where the target appeared unilaterally in the visual screen; (ii) *bilateral*, in which the target was presented bilaterally; (iii) *neutral incongruent*, where the target was presented unilaterally along with a face showing a neutral (non-emotional) expression; and (iv) *emotional incongruent,* where the target was presented along with a face showing a contrasted emotional expression. Facial images of the emotions were always displayed at the highest (100%) intensity level. Participants had to respond as soon as they noticed the target (regardless of its position or number) on the screen, pushing the space bar on the keyboard with the dominant (right) hand. Fear and anger were studied independently in different blocks. At the beginning of each block, the target emotion was verbally declared by the experimenter. Stimuli stayed for a duration of 250 ms. Participants had a maximum of 1,500 ms to provide an answer. The inter-stimulus interval varied randomly between 650 and 950 ms. For each condition (unilateral; congruent bilateral; incongruent emotional–emotional condition; incongruent neutral–emotional condition), 32 valid trials and 16 catch trials were shown in random order in four blocks. The block-order was reversely counterbalanced (i.e., ABBA order) to balance the order and sequence effects within subjects: half of the participants received the order ABBA: anger, fear, fear, anger; the other half recieved the opposite order BAAB: fear, anger, anger, fear. Overall, 768 trials were administered. There was a short break (2 min) between each block. Two main experimental outcomes were tested: the *Reaction Time* in milliseconds from stimulus onset relative to valid trials, representing the level of detection, and the percentage of *Accuracy* (% hits – % false alarms), representing the level of recognition.

### Analyses

An independent sample t-test was used to assess any differences between the two groups (participants with AN vs. controls) relative to the demographical characteristics (age and education) and the level of BMI. Furthermore, an independent sample t-test was used to assess any differences between the two groups (participants with AN vs. controls) in the subscores and the total score reported on the Toronto Alexithymia Scale - 20. For the facial emotion recognition task, we followed the same rationale as previous studies (Scarpina et al., 2018, 2021, 2022) about preprocessing data as well as data analyses. The two experimental outcomes of reaction time and level of accuracy were independently analyzed through a mixed ANOVA with the within-subjects factors of *Condition* (unilateral, bilateral, neutral incongruent, and emotional incongruent) and the between-subjects factor of *Group* (participants with AN vs. controls). Bonferroni-estimated marginal mean comparisons were applied as post-hoc analyses when the main effect of *Condition* or the interactions were significant. In case of the significant main effect of the between-subjects *Group* or its significant interaction with the experimental between-subjects factors, we planned to run the main analysis again, introducing the global score at the Toronto Alexithymia Scale - 20 as a covariate. This analysis allowed us to verify if the main behavioral difference between our groups (if any) could be explained by the individual level of alexithymia.

### Sample size calculation

Overall, 36 participants, split into two equal-sized groups of n = 18, should be enrolled to achieve a power of 0.95, considering a medium effect size (d = 0.25) and a two-tailed alpha of 0.05. This analysis was performed using G*Power3 (Faul et al., 2007).

## Results

### Participants

Eighteen women with AN (age in years M = 28.11; SD = 12.29; range = 18–54, Education in years M = 12.83; SD = 3.51; range = 8–18, BMI M = 15.16; SD = 1.9; range = 12.17–18.18) were enrolled.

Half of the participants (N = 9; age in years M = 26.44; SD = 12.39; range = 18–54, education in years M = 13.11; SD = 3.4; range = 8–18, BMI M = 13.96; SD = 1.02; range = 12.17–15.15) were classified as restrictive; the other half (N = 9; age in years M = 29.66; SD = 12.79; range = 19–54, education in years M = 13.11; SD = 3.4; range = 8–18, BMI M = 17.47; SD = 2.48; range = 13.96–22.46 as binge eating/purging type.

In Table 1, details about the clinical descriptors of our participants with AN are fully reported.

**Figure 1:**
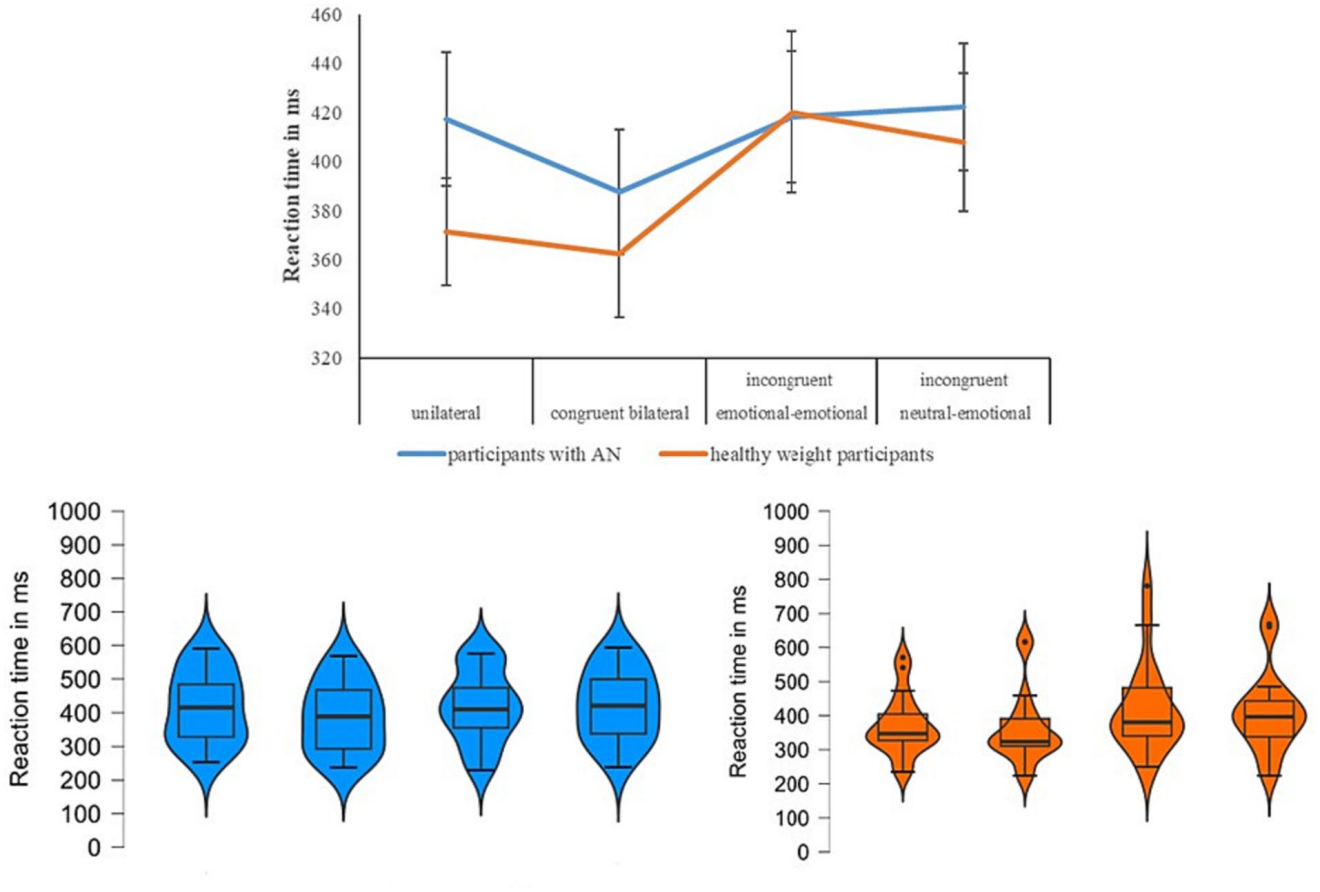
For each experimental condition (x-axis), in the upper panel, we show the mean relative to the reaction time expressed in milliseconds on the y-axis for the two groups (participants with anorexia nervosa in blue vs. healthy weight participants in orange) when the emotion of fear was tested. In the below panel, we show the violin plots (on the left for the group participants with anorexia nervosa, on the right for the healthy participants), and specifically the median (horizontal dark black line), the interquartile range (the vertical line), the shape of the distribution, and the outliers (dark circle).

**Table 1:** Mean, standard deviation, and range relative to the scores at the questionnaires about eating behavior (Binge Eating Scale and Eating Disorders Inventory-3), symptoms of psychopathology (Symptom Checklist-90), and overall psychological well-being (Psychological General Wellbeing Index) in participants affected by anorexia nervosa.

	Mean	Standard deviation	Min	Max
**Binge eating scale**				
Score	33.53	19.92	10	98
**Eating disorder inventory™–3**				
Drive for Thinness	24.35	6.13	5	28
Bulimia	9.71	10.10	0	31
Body Dissatisfaction	29.47	6.97	16	40
*Eating Disorder Risk*	63.53	13.72	32	86
Low Self-Esteem	16.88	5.37	5	24
Personal Alienation	15.41	6.11	6	26
Interpersonal Insecurity	12.35	6.11	2	26
Interpersonal Alienation	13.29	5.01	1	22
Interoceptive Deficits	24.53	8.29	10	34
Emotional Dysregulation	11.71	6.76	0	21
Perfectionism	12.12	5.18	2	22
Asceticism	16.00	5.93	7	26
Maturity Fears	15.82	7.74	6	30
Ineffectiveness	32.29	10.85	11	50
Interpersonal Problems	25.65	9.94	5	44
Affective Problems	36.24	14.32	10	54
Overcontrol	28.12	9.81	9	46
General Psychological Maladjustment	138.12	41.72	61	191
**Symptom Checklist-90**				
Somatization	1.78	1.12	0.08	3.92
Obsession-compulsion	2.38	0.97	0.7	3.6
Interpersonal sensitivity	2.48	0.78	1.33	3.89
Depression	2.83	0.82	0.92	3.77
Anxiety	2.36	0.83	1	3.7
Hostility	2.54	5.16	0	22.33
Phobic anxiety	1.17	0.84	0	2.71
Paranoid ideation	1.63	0.65	0.5	2.5
Psychoticism	2.18	0.85	0.33	3.17
Total score	2.12	0.71	0.78	3.03
**Psychological general well being index**				
Anxiety	8.24	3.99	1	14
Depression	5.24	4.18	0	13
Positive well-being	3.82	3.00	0	9
Self-control	6.00	3.45	1	13
General healthy	7.65	3.48	1	14
Vitality	6.35	4.77	1	15
Total score	37.29	17.65	6	70

From the previous database (Scarpina et al., 2018, 2021, 2022), we extracted the information relative to 18 healthy individuals (age in years M = 46.44; SD = 11.26; range = 23–59; education in years M = 14.55; SD = 2.87; range = 8–18). For this group, the mean BMI was 22.26 (SD = 1.31; range = 20–24.91).

Participants with AN were significantly younger than controls [*t* (34) = 4.66; *p* < 0.001; d’ = 1.55]; the two groups were comparable about the level of education [*t* (34) = 1.52; *p* = 0.13; d’ = 0.53]. As expected, the participants with AN reported a significantly lower level of BMI than controls [*t* (34) = 13.05; *p* < 0.001; d’ = 7.06].

### The level of alexithymic traits

Scores and statistical differences relative to the Toronto Alexithymia Scale - 20 between our participants with AN and healthy controls are reported in Table 2. As expected, a higher expression of alexithymic traits in our participants with AN in comparison with controls was observed.

**Table 2:** Mean, standard deviation, and range relative to the three subscores and the global score of the Toronto Alexithymia Scale - 20 are shown for the participants with anorexia nervosa and the healthy weight participants.

	Participants with AN	Healthy weight participants	Statistical differences
Difficulties in identifying feelings	M = 25.62; SD = 6.3; range = 12–33	M = 12.44; SD = 4.25; range = 7–21	*t* = 7.34; *p* < 0.001; d’ = 2.45 *
Difficulties in describing feelings	M = 18.72; SD = 4.62; range = 9–25	M = 10.88; SD = 4.33; range = 4–19	*t* = 5.24; *p* < 0.001; d’ = 1.75 *
Externally-oriented thinking	M = 21.44; SD = 6.37; range = 13–35	M = 14.61; SD = 6.33; range = 4–27	*t* = 3.26; *p* = 0.003; d’ = 1.07 *
Global score	M = 65.77; SD = 13.93; range = 37–87	M = 37.94; SD = 12.83; range = 15–59	*t* = 6.23; *p* < 0.001; d’ = 2.07 *

Statistical results are also reported, with * indicating significant differences.

df = 34.

According to some evidence [i.e., (Pasini et al., 1992; Mattila et al., 2006), the expression of alexithymia may be linked to age, especially in the elderly. According to the demographic information, our healthy weight participants were significantly older than participants with AN; even the range mainly overlapped, and none of our participants were in the category of older adults (65 and older). Nevertheless, to clarify our result, we performed a supplementary analysis in which we compared the scores of the two groups on the Toronto Alexithymia Scale - 20, including the factor *Age* as a covariate. According to this analysis, we confirmed that the two groups reported different scores in terms of difficulties in identifying feelings [*F* (1,33) = 29.39; *p* = 0.001; η^2^ = 0.47], while the covariate was not significant [*F* (1,22) = 4.31; *p* = 0.14; η^2^ = 0.7]. The main effect of *Group* was confirmed also in the case of the score measuring the difficulties in describing feelings [*F* (1,33) = 8.96; *p* = 0.005; η^2^ = 0.214], and the covariate was significant [F (1,33) = 4.72; *p* = 0.04; η^2^ = 0.15]. We did not observe the significant difference between groups in terms of externally-oriented thinking [*F* (1,33) = 3.35; *p* = 0.07; η^2^ = 0.09], and the covariate was not significant [F (1,33) = 1.25; *p* = 0.27; η^2^ = 0.03]. Finally, when we considered the total score, which will eventually be included in the main statistical model about the experimental data, we again observed the significant main difference between groups [F (1,33) = 16.36; *p* < 0.001; η^2^ = 0.33], but the covariate was not significant [*F* (1,33) = 1.85; *p* = 0.18; η^2^ = 0.05]. This result suggested that, overall, the different levels of expression of alexithymic traits observed between our participants with AN and healthy weight participants were not linked to age.

### Facial emotion recognition task

Because of the preprocessing data, the 0.3% of answers relative to the emotion of fear and 0.21% for the emotion of anger provided by the group of participants with AN were not included in the analysis since they were anticipations. Similarly, the 0.99% of answers relative to the emotion of fear and the 0.05% relative to anger provided by controls were not included in the analysis.

### Fear

Behavioral data are reported in Table 3.

**Table 3:** About the emotion of fear, mean (M), standard deviation (SD), and range for each experimental condition (unilateral, congruent bilateral, incongruent emotional- emotional, incongruent neutral–emotional) relative to the two groups (participants with anorexia nervosa vs. controls) are reported for the reaction time (expressed in milliseconds) and the level of accuracy (expressed in percentage).

		unilateral	congruent bilateral	incongruent bilateral	
				emotional–emotional	neutral–emotional
Reaction time in milliseconds					
participants with AN	M	417	388	418	422
	SD	116	108	114	110
	range	223–660	217–108	197–636	204–614
healthy weight participants	M	371	363	420	408
	SD	93	111	140	120
	range	218–586	194–111	227–884	215–743

Level of accuracy in percentage					
participants with AN	M	53.99	56.78	30.98	31.52
	SD	22.47	21.53	19.93	20.51
	range	6.94–88.89	21.18–21.53	−13.89–66.67	−11.81–67.36
healthy weight participants	M	66.88	67.03	44.66	46.43
	SD	15.50	15.80	20.16	20.83
	range	12.15–85.76	9.03–15.80	0–83.33	−2.78–86.11

RT. According to the Shapiro–Wilk test, the RT relative to the within-subjects factor of Condition followed a normal distribution [*p* ≥ 0.08], except for the data of the congruent bilateral [W (18) = 0.84, *p* = 0.003] and the emotional–emotional [W (18) = 0.86, *p* = 0.015] conditions in the healthy weight group. The main effect of Condition [F (3,102) = 11.5; *p* < 0.001; η^2^ = 0.25] was significant: in the congruent bilateral condition, all participants reported lower reaction time (i.e., were faster) when compared with the other conditions [p < 0.035]. Moreover, significant higher RTs (i.e., were slower) were reported in the incongruent neutral–emotional condition in comparison with unilateral [*p* = 0.42] and congruent bilateral [*p* < 0.001], but it was similar with the incongruent emotional–emotional condition [*p* = 1]. Overall, this performance was in line with the redundant target effect. The main effect of Group was not significant [F (1,34) = 0.35; *p* = 0.55; η^2^ = 0.01] (participants with AN = 411; SD = 111; controls M = 390; SD = 118). To better understand this result, we computed the Bayes factor using the software JASP (JASP Team, 2019) to classify the strength of the evidence (Jeffreys, 1939; Kass and Raftery, 1995; Dienes, 2014). Specifically, this computation was performed to test whether the non-significant result supports a null hypothesis over a theory or whether the data are just insensitive (Dienes, 2014). The Bayes factor confirmed extreme evidence for the null hypothesis (BF10 < 0.001) (Lee and Wagenmakers, 2014). Crucially, we observed a significant interaction Condition*Group [F (3,102) = 2.81; *p* = 0.043; η^2^ = 0.07]: about controls, we observed a performance in line with the redundant target effect, with no significant difference between unilateral and congruent bilateral [p = 1] conditions and between the two incongruent conditions [*p* = 1], while all the other comparisons were significant [p ≤ 0.027]. Instead, in the participants with AN, we did not observe the redundant target effect: they reported a significantly lower reaction time (i.e., a faster performance) in the congruent bilateral (which is the easist condition) when compared with the unilateral [*p* = 0.018] and the incongruent neutral–emotional [*p* = 0.011] conditions, but with no other significant differences [*p* ≥ 0.9] (Figure 1).

### RT

According to Shapiro-Wilk test, the RT relative the within-subjects factor of Condition followed a normal distribution [p≥0.08], except for the data of the congruent bilateral [W(18)=0.84, p=0.003] and the emotional-emotional [W(18)=0.86, p=0.015] conditions in healthy weight group. The main effect of Condition [F(3,102)=11.5; p<0.001; η2=0.25] was significant: in the congruent bilateral condition all participants reported lower reaction time (i.e., were faster) when compared with the other conditions [p<0.035]. Moreover, significant higher RTs (i.e., were slower) were reported in the incongruent neutral-emotional condition in comparison with unilateral [p=0.42] and congruent bilateral [p<0.001], but it was similar with the incongruent emotional-emotional condition [p=1]. Overall, this performance was in line with the redundant target effect. The main effect of Group was not significant [F(1,34)=0.35; p=0.55; η2=0.01] (participants with AN=411; SD=111; controls M=390; SD=118). To better understand this result, we computed the Bayes factor using the software JASP [50] to classify the strength of evidence [51-53]. Specifically, this computation was performed to test whether the non-significant result supports a null hypothesis over a theory, or whether the data are just insensitive [53]. The Bayes factor confirmed extreme evidence for the null hypothesis (BF10 < 0.001) [54]. Crucially, we observed a significant interaction Condition*Group [F(3,102)=2.81;p=0.043; η2=0.07]: about controls, we observed a performance in line with the redundant target effect, with no significant difference between unilateral and congruent bilateral [p=1] conditions and between the two incongruent conditions [p=1], while all the other comparisons were significant [p≤0.027]. Instead, in the participants with AN, we did not observe the redundat target effect: they reported a significant lower reaction time (i.e., a faster performance) in the congruent bilateral (which is the easist condition) when compared with the unilateral [p=0.018] and the incongruent neutral-emotional [p=0.011] conditions, but with no other significant differences [p≥0.9] (Figure 1).

### Accuracy

According to the Shapiro–Wilk test, the level of accuracy relative to the within-subjects factor of *Condition* followed a normal distribution [*p* ≥ 0.31], except for the data relative to the unilateral [W (18) = 0.84; *p* = 0.006] and congruent bilateral condition [W (18) = 0.82, *p* = 0.003] in healthy weight participants. We observed the significant main effect of *Condition* [*F* (3,102) = 111.34; *p* < 0.001; η^2^ = 0.76]: the behavioral results were in line with the redundant target effect, since a higher level of accuracy emerged in the unilateral and congruent conditions when compared with both the incongruent conditions [*p* always ≤0.001], with no difference within them [p always = 1]. We observed a significant main effect of *Group* [*F* (1,34) = 4.93; *p* = 0.03; η^2^ = 0.12]: indeed, participants with AN (M = 43.31; SD = 24.18) reported a lower level of accuracy than controls (M = 56.25; SD = 21). We did not observe a significant interaction Condition*Group [*F* (3,102) = 0.62; *p* = 0.6; η^2^ = 0.01]; this result was confirmed by the Bayes factor (BF10 = 0.003) (Lee and Wagenmakers, 2014). Considering the results, we ran the analysis again, introducing the covariate, meaning the total score on the Toronto Alexithymia Scale - 20. The covariate was significant [F (1,33) = 9,06; p = 0.005; η^2^ = 0.21]. We confirmed the main effect of *Condition* [F (3,99) = 4.009; *p* = 0.01; η^2^ = 0.1] Notably, the main effect of *Group* was not anymore significant [F (1,33) = 0.26; *p* = 0.61; η^2^ = 0.008]. As observed for the reaction time, also the between-subjects differences in the level of accuracy may be related to the individual’s different levels of alexithymic traits.

Considering the results, and specifically the significant interaction Condition*Group, we investigated the role of alexithymia on the performance. We performed the main analysis again, including the global score reported at the Toronto Alexithymia Scale - 20 as a covariate. The covariate was significant [*F* (1,33) = 5.69; *p* = 0.02; η^2^ = 0.14]. The experimental main effect of *Condition* was still significant [*F* (3,99) = 2.75; p = 0.04; η^2^ = 0.07]. Moreover, the main effect of *Group* was not significant [F (1,33) = 1.71; p = 0.2; η^2^ = 0.04]. However, we did not observe the interaction Condition*Group as significant [F (1,33) = 0.58; *p* = 0.44; η^2^ = 0.01]. This result crucially suggests that the different behavioral effects observed in the main task may be related to the individual’s different levels of alexithymic traits.

Considering that our groups were significantly different in terms of age, we ran the main statistical model again, including *Age* as a covariate. When we analyzed the data relative to the reaction times, we observed that the covariate was not significant [F (1,33) = 1.6; *p* = 0.214; η^2^ = 0.04], while we confirmed the main effect of *Condition* [F (3,99) = 5.03; p = 0.003; η^2^ = 0.13] and the interaction Condition*Group [F (3,99) = 4.9; p = 0.003; η^2^ = 0.12] reported in the main analysis. Similarly, when we analyzed the data relative to the level of accuracy, again the covariate was not significant [F (1,33) = 1.01; *p* = 0.32; η^2^ = 0.03], while we confirmed the significant main effect of *Condition* [F (3,99) = 11.98; *p* < 0.001; η^2^ = 0.26] and the main effect of *Group* [F (3,99) = 5.58; *p* = 0.024; η^2^ = 0.14] reported in the main analysis. Overall, these supplementary analyses pointed out that the different performance between the two groups in the main experimental task in terms of velocity and level of accuracy was not linked to age.

### Anger

Behavioral data are reported in Table 4.

**Table 4:** About the emotion of anger, mean (M), standard deviation (SD), and range for each experimental condition (unilateral, congruent bilateral, incongruent emotional–emotional, incongruent neutral–emotional) relative to the two groups (participants with anorexia nervosa vs. controls) are reported about the reaction time (expressed in milliseconds) and the level of accuracy (expressed in percentage).

		unilateral	congruent bilateral	incongruent bilateral	
				emotional–emotional	neutral–emotional
Reaction time in milliseconds					
participants with AN	M	407	389	399	400
	SD	96	96	101	91
	range	244–610	224–96	204–613	233–598
healthy weight participants	M	396	383	418	414
	SD	140	131	137	114
	range	212–830	212–131	190–887	253–719
Level of accuracy in percentage					
participants with AN	M	48.61	53.00	27.51	22.57
	SD	24.43	25.71	18.61	20.43
	range	−1.74– 85.76	−0.35– 25.71	−10.42– 56.94	−27.08– 50.00
healthy weight participants	M	61.22	58.92	37.89	32.95
	SD	18.15	17.12	19.55	20.42
	range	24.65–86.11	15.63–17.12	−11.81–75.00	−20.14–80.56

### RT

According to the Shapiro–Wilk test, the reaction times relative to the within-subjects factor of *Condition* followed a normal distribution [*p* ≥ 0.063], except for the data relative to the unilateral condition [W (18) = 0.85, *p* = 0.012] in the healthy weight group. We observed a significant main effect of *Condition F* (3,102) = 2.87; p = 0.03; η^2^ = 0.07]: specifically, all participants reported significantly higher RTs for the congruent bilateral condition in comparisons with both incongruent emotional–emotional [*p* = 0.05] and incongruent neutral–emotional [p = 0.01] conditions, with no other significant differences [*p* ≥ 0.67]. The main effect of *Group* was not significant [*F* (1,34) = 0.014; *p* = 0.9; η^2^ < 0.001]: participants with AN (M = 398; SD = 95) reported similar RT than controls (M = 402; SD = 130). We underlined that the Bayes factor (BF10 = 0.65) suggested anecdotal evidence for the null hypothesis (Lee and Wagenmakers, 2014). The interactions with *Condition* [F (3,102) = 1.42; *p* = 0.23; η^2^ = 0.04] were not significant.

### Accuracy

According to the Shapiro–Wilk test, the level of accuracy relative to the within-subjects factor of *Condition* followed a normal distribution [*p* ≥ 0.26]. The main effect of *Condition* was significant [F (3,102) = 153.28; p < 0.001; η2 = 0.81]: all individuals reported no different level of accuracy between the unilateral and congruent bilateral conditions [*p* = 1], while all the other differences between the conditions were significant [*p* ≤ 0.001]. No significant main effect of *Group* (participants with AN M = 37.92; SD = 25.83; controls M = 47.74; SD = 22.46) [F (1,34) = 2.84; *p* = 0.1; η^2^ = 0.07] was observed. This pattern of the results was confirmed by the computation of the Bayes factor (i.e., extreme evidence for the null hypothesis, BF10 < 0.001) (Lee and Wagenmakers, 2014). Moreover, no significant interaction of the factor *Group* with the experimental factors of *Conditions* [F (3,102) = 1.39; *p* = 0.24; η^2^ = 0.03] emerged.

Since we did not observe any altered behavior of participants with AN in detecting and recognizing anger facial expressions when compared with controls, no further analysis was done.

## Discussions

This study aimed to test the ability to recognize fearful and angry facial expressions when tested through implicit behavior (Scarpina et al., 2018, 2021, 2022) in women with AN compared with healthy weight women, taking into account the individual expression of alexithymic traits.

We observed significantly altered behavior in our participants with AN when the emotion of fear, but not anger, was tested. In detail, in terms of detection (i.e., the reaction time), our participants with AN had a behavioral advantage (i.e., being faster) only in the congruent bilateral condition, which is the easiest experimental one since two identical faces with the same expressions of fear were shown. However, in all the other tested experimental conditions, they were slower, and globally, they did not show a performance in line with the expected experimental phenomenon of the redundant target effect. This result may be read in the direction of a very pervasive alteration in detecting fearful expressions. The performance was similarly altered when it was scored in terms of the level of accuracy: individuals with AN were overall less accurate compared with healthy weight controls, independently from the tested conditions. Crucially, the altered performance emerged only in the case of fearful expressions; indeed, we did not observe a difference between our participants with AN and the healthy weight controls for angry facial expressions. Notably, the performance of affected participants in the case of angry facial expressions was in line with the redundant target effect.

This may be a very interesting result: the alterations in emotional processing, at least at the level of facial recognition, in AN seemed to be emotion-related, with the highest difficulties for the emotion of fear. Our results, which are in line with some previous evidence (Kucharska-Pietura et al., 2004; Lulé et al., 2014), might not come as a surprise if they are read in the context of the theoretical models for basic emotions: each emotion responds to specific triggers with distinct adaptive functions (Ekman and Friesen, 1976; Izard and Haynes, 1988; Elkman, 1992; Panksepp, 1998; Izard et al., 1999). Thus, some individuals may show difficulties with some emotions but not with others. As humans, we experience different emotional experiences for different triggers. We feel fear when we perceive something or someone as a threat; suddenly, we have to mobilize our resources to cope with the danger. Instead, we feel anger when something or someone prevents us from accomplishing our desires, needs, or goals; then, our efforts are directed to overcome the obstacle, or—if it is not possible—to deal with the feelings of frustration and powerlessness. According to Fox and Power (Fox and Power, 2009), both the emotions of fear and anger may be altered in eating disorders, but with some differences. The alteration of fear processing would be more related to the individual experience and specifically to the manifestation of anxiety symptoms. On the other hand, the alteration of anger processing would be linked to a higher level of inhibition in emotional expression to avoid confrontations and conflicts with others. Thus, anger may refer more to an inter-individual dimension of emotional experience. This phenomenological difference may mirror the cognitive process implied by the facial emotion recognition task. First, the primary visuo-perceptual processing of faces is enhanced; only successively, a conceptual analysis of the emotion conveyed by the face (Adolphs, 2002) emerges. Nevertheless, both processes emerge hierarchically before any further higher-cognitive processes, which may be more related to the complex interpersonal dimension, such as who is showing the emotion and what is presumably his/her intention.

A second crucial result emerged from our study: the altered performance in processing fear when expressed by human faces in our participants with AN was related to the higher level of alexithymic traits. This result was in disagreement with the two previous studies (Kessler et al., 2006; Lulé et al., 2014), which reported no significant relationship between facial emotion recognition ability and levels of alexithymia in this clinical population. However, different results across studies may be explained by the different methodological and statistical approaches. Nevertheless, our results reconciled with the very large amount of evidence describing the alteration in facial emotion recognition as related to higher expressions of alexithymic traits [i.e., 23–25]. Interestingly, this link could be explained by difficulties in terms of emotional embodiment (Parker et al., 1993; Scarpazza et al., 2015; Maier et al., 2016; Scarpazza et al., 2018); in other words, altered abilities in decoding sensory input informing about the physiological state of the body (i.e., interoception (Craig, 2003)), as well as when experiencing emotions (Damasio, 1994). Such a difficulty may emerge more in the case of fear and anxiety-related symptoms (Craig, 2002; Paulus and Stein, 2006), about which several bodily signals can be decoded wrongly, such as tachycardia, rapid breathing, and increased sweating. Preliminary evidence about altered interoception in AN (Pollatos et al., 2008; Kerr et al., 2016; Kinnaird et al., 2020), also linked with the high rates of alexithymia (Jacquemot and Park, 2020) has been reported in the literature. Thus, individuals with AN and associated higher expressions of alexithymic traits may misinterpret bodily sensations due to the recognition of fearful expressions in others, decreasing their ability to process and cope with this emotion. Our hypothesis can be read in the framework of the motivational theories of emotions (Bradley et al., 2001; Lang and Bradley, 2010): expressed emotions are founded on motivational cerebral circuits, which are functionally engaged in the case of appetitive and aversive environmental and memorial cues, mediating appetitive and defensive reflexes that tune sensory systems and mobilize the organism for action and underly negative and positive effects. The hypothesis, which requires future investigation in both neuropsychology and clinical psychology, through neurophysiological measures compared with explicit subjective reports about individual levels of arousal generated by emotional stimuli, would revitalize treatments for psychological difficulties in AN, posing the bodily experience even in terms of sensory signals (i.e., “*What I feel? How I feel*?”) at the center of the individual’s narration about emotions. Our evidence relative to the altered performance in the case of fearful expressions in AN as linked to the higher level of alexithymia may also direct psychological interventions. There are several lines of evidence suggesting a complex role for alexithymia in influencing psychiatric treatment outcomes (consult (Pinna et al., 2020) for a systematic review). Cameron et al. (Cameron et al., 2014) observed a significant reduction in alexithymia scores following treatments only when they directly targeted alexithymic symptoms, whereas studies in which psychological interventions did not specifically target alexithymia reported more inconsistent results. Focusing on AN, studies reported in the literature seem in agreement in suggesting that the alexithymia levels remain high (even if reduced) after specific treatment [i.e., (Gramaglia et al., 2020; Meneguzzo et al., 2022). Then, in consideration of this evidence, clinicians may aim to support patients with AN in increasing their levels of emotional awareness (i.e., the explicit ability to identify and describe one’s own emotions and other people’s emotions (Lane and Schwartz, 1987) in order to be more efficient in emotional regulation and information processing (Szczygieł et al., 2012), especially focusing on the emotion of fear.

As noted by Calbi et al. (2017), most of the evidence about emotional processing in psychopathology is based on the detection and recognition of emotions shown by isolated facial expressions, as in this study. Indeed, the recognition of others’ emotions via facial expressions has a survival role for humans, sustaining efficient complex cognitive functions and processes, such as communication, empathy, and social cognition (Ekman, 1992): Because of its crucial role in human experience, facial emotion recognition is extensively investigated in the psychological sciences in healthy individuals as well as in clinical conditions (Vaioli and Scarpina, 2021). However, it should be underlined that in facial emotion recognition tasks, participants have to recognize and detect the presence of an emotional target contrasted with emotional and neutral distractors, in the absence of any other contextual information. Thus, this task cannot provide further information about emotional processing. Some research pointed out the role of contextual cues on the evaluation of facial expressions of emotion [i.e., (Mirabella, 2018; Moors and Fischer, 2019; Mancini et al., 2020, 2022; Calbi et al., 2022). Specifically, according to the appraisal theories, emotions are elicited by the subjective evaluation (appraisals) of events and situations (Roseman and Smith, 2001), including others’ reactions: individuals evaluate an emotional event while being affected by the way in which others evaluate and feel about the same event (Manstead and Fischer, 2001), especially in ambiguous situations (Fischer et al., 2004). In other words, the appraisal of emotional valence is not automatic; it depends on the relevance of the emotional stimuli for individual aims. For example, Mirabella et al. (2023) investigated the effect of facial emotional expressions (and specifically of the emotions of anger and happiness) on forward gait initiation. Mirabella (2018) tested a go/no-go task in which participants were instructed to perform or inhibit reaching arm movements in response to fearful or happy facial expressions. Focusing on the social dimension, Mumenthaler and Sander (Mumenthaler and Sander, 2012) tested the recognition of dynamic facial expressions of emotion when the target was shown contextually with faces expressing primary or neutral emotion and looked (or not) at the target. In other words, in this experiment, gaze direction was used as a social contextual cue. The adoption of tasks as described may be useful in the context of AN, even with some cautions in the case of actions, as in Fischer et al. (2004); Mirabella (2018): multiple evidence suggest alterations in imagined (Guardia et al., 2010, 2012; Metral et al., 2014; Beckmann et al., 2021; Scarpina et al., 2022) and real (Guardia et al., 2010, 2012; Keizer et al., 2013; Metral et al., 2014) bodily movements in AN. Difficulties in socio-emotional functioning are generally described in this clinical population [i.e., (Noordenbos, 2011; Treasure and Schmidt, 2013). As reported by Oldershaw et al. (2011), affected individuals have not only altered facial emotion recognition ability, as testified by our results, but also difficulties in the acquisition of social-affective values and responses, and specifically heightened sensitivity and bias toward or avoidance of ‘threatening’ emotional stimuli. Could emotions be processed differently when they are contextual, and specifically action-related? Future investigations may explore this question.

This study suffered from some criticism. The sample size was limited. Moreover, we did not collect healthy controls *ad-hoc* for this cross-sectional study, while we extracted the data from previous studies (Scarpina et al., 2018, 2021, 2022). Because of this, further details about the psychological functioning of these participants cannot be reported. Nevertheless, when clinical questionnaires as used in this research for the psychopathological description of our participants in AN are used in the case of healthy individuals, a floor effect, i.e., a considerable percentage of participants obtain the minimum available score, is generally observed. As done also in the previous studies (Kucharska-Pietura et al., 2004; Mendlewicz et al., 2005; Kessler et al., 2006; Castro et al., 2010; Lulé et al., 2014; Dapelo et al., 2016; Gramaglia et al., 2016; Sfärlea et al., 2016; Wyssen et al., 2019; Blomberg et al., 2021; Duriez et al., 2021), participants with both types of AN (restrictive vs. binge eating/purging type) were included in the same sample. Since this factor was not included in our statistical model as a between-subjects factor, we cannot perform a-posteriori this analysis, since it would be underpowered. However, in future, it would be of some interest to compare the behavioral responses to the emotional tasks between the different types of eating disorders. Weinbach et al. (2018) reported that even if both restrictive and binge eating/purging eating disorders (thus, not specifically in the context of AN) have been associated with emotion regulation difficulties, on the other hand, the presence of binge eating or purging episodes may be linked with a higher difficulty in emotion regulation. If such a difference may emerge also in the context of the detection and recognition of others’ facial expressions in AN, it remains to be determined.

## Data availability statement

The datasets presented in this study can be found in online repositories. The names of the repository/repositories and accession number(s) can be found at: https://doi.org/10.5281/zenodo.8126052.

## Ethics statement

The studies involving humans were approved by Ethical Commission of Istituto Auxologico Italiano. The studies were conducted in accordance with the local legislation and institutional requirements. The participants provided their written informed consent to participate in this study.

## Author contributions

GV: Conceptualization, Data curation, Investigation, Writing – original draft. IB: Data curation, Investigation, Writing – review & editing. VV: Data curation, Investigation, Writing – review & editing. LM: Supervision, Writing – review & editing. GC: Supervision, Writing – review & editing. AM: Funding acquisition, Supervision, Writing – review & editing. FS: Conceptualization, Formal analysis, Investigation, Methodology, Writing – original draft.
